# Challenges in Diagnosis and Treatment of Achalasia Cardia in Uganda: A Case Report of an Adolescent Female Presenting With Dysphagia

**DOI:** 10.1155/cris/5527940

**Published:** 2025-03-11

**Authors:** Tracy Tushabe Namata, Deogratius Bakulumpagi, Anna Nyisomeh, Davis Nsamba, Brian Bbosa, Didas Mugisa

**Affiliations:** ^1^Department of Surgery, St. Francis Hospital Nsambya, Kampala, Uganda; ^2^Department of Surgery, Mulago National Referral Hospital, Kampala, Uganda; ^3^Head of Cardiothoracic Surgery, St. Francis Hospital Nsambya, Kampala, Uganda

**Keywords:** achalasia cardia, adolescent, dysphagia, esophageal perforation

## Abstract

**Background:** Our case highlights the challenges in diagnosing and managing achalasia cardia, particularly in resource-limited settings and more so in adolescents who fall outside of the typical age range.

**Case Presentation:** We present a case of an 18-year-old female from Uganda who was admitted with a 6-month history of progressive dysphagia, weight loss, and postprandial vomiting. Diagnosis of achalasia cardia was confirmed via endoscopy and barium swallow. Heller cardiomyotomy via open transthoracic approach was performed, but she developed an esophageal perforation, which was successfully managed with repeat thoracotomy and esophageal repair. Complete resolution of achalasia symptoms was achieved at a 5-month follow-up.

**Conclusion:** This case highlights the importance of maintaining a high index of clinical suspicion, especially in young patients, and the significance of informed consent prior to initiating treatment. Additionally, it emphasizes the importance of early recognition of treatment-related complications, such as esophageal perforation, as key to prompt management and improved patient outcomes.

## 1. Introduction

Achalasia cardia is an esophageal motility disorder resulting from failure of lower esophageal sphincter (LES) relaxation. The exact pathogenesis of achalasia cardia is unclear; however, some evidence suggests neuronal degeneration, autoimmunity, or infectious etiology [1–3].

This disorder is relatively rare with an incidence rate of ~1/100,000 people per year which increases with age, and it mainly affects adults aged 25–60 years [2], with the minority of cases seen among children and adolescents [2, 3].

Achalasia cardia presents with a wide range of symptoms including dysphagia, heartburn, and postprandial vomiting among others [1–4]. In adolescents and young adults, it is commonly misdiagnosed as gastroesophageal reflux disease or anorexia nervosa leading to delays in diagnosis [3, 4].

Treatment options include pharmacologic therapy and surgery, which may be laparoscopic or open surgery [5]. Esophageal perforation is a recognized but very rare complication of surgery for achalasia cardia [6]; therefore, informed consent should be obtained prior to surgery. Early identification of this complication is vital to reduce the associated morbidity and mortality.

In this report, we present a challenging case of an adolescent female brought to our surgical service at St. Francis Hospital Nsambya, Uganda, and we discuss the clinical presentation, diagnostic workup, and treatment complications encountered during her care.

## 2. Case Presentation

### 2.1. Case History and Examination

In May 2023, we received an 18-year-old female at our surgical service at St. Francis Hospital Nsambya, Kampala, Uganda, as a referral from a community hospital in Eastern Uganda for further management. She presented with a 6-month history of progressive dysphagia for both solids and liquids. Initially, she was able to feed on a very soft diet with ample fluids to aid swallowing. However, 2 months prior to admission to our service, she began experiencing complete difficulty in swallowing both solids and liquids. This was accompanied by postprandial vomiting, with vomitus predominantly consisting of ingested undigested food particles, and retrosternal chest pain that was burning in nature. Additionally, she reported a marked weight loss of about 20 kg observed over the same period. There was no reported history of self-induced vomiting or other behaviors that interfere with weight gain. The patient had no known chronic illnesses, no history of caustic ingestion or travel to areas endemic with *Trypanosoma cruzi*, and no familial swallowing problems.

On admission, she was sick-looking, very weak, severely wasted, and severely dehydrated. Her weight was 37.0 kg, height was 157 cm, and BMI was 15.0 kg/m^2^. She was hypotensive with blood pressure of 90/50 mmHg and tachycardia of 115 beats per minute. Her body temperature and oxygen saturation were normal. Her abdomen was scaphoid, soft, and nontender, with no masses felt on palpation. The rest of the examination findings were unremarkable.

### 2.2. Differential Diagnosis

At the time of admission, the following differential diagnoses were considered: achalasia cardia, gastroesophageal reflux disease, esophageal webs, esophageal stricture, and diffuse esophageal spasm. However, investigations confirmed the diagnosis of achalasia cardia and also ruled out other possibilities. Anorexia nervosa was excluded based on clinical history and consultation with a clinical psychologist.

### 2.3. Investigations

On admission, laboratory investigations revealed moderate hypokalemia of 2.7 mmol/L, moderate anemia of 9.5 g/dL, normal random blood glucose, and renal function test results.

**Figure 1:**
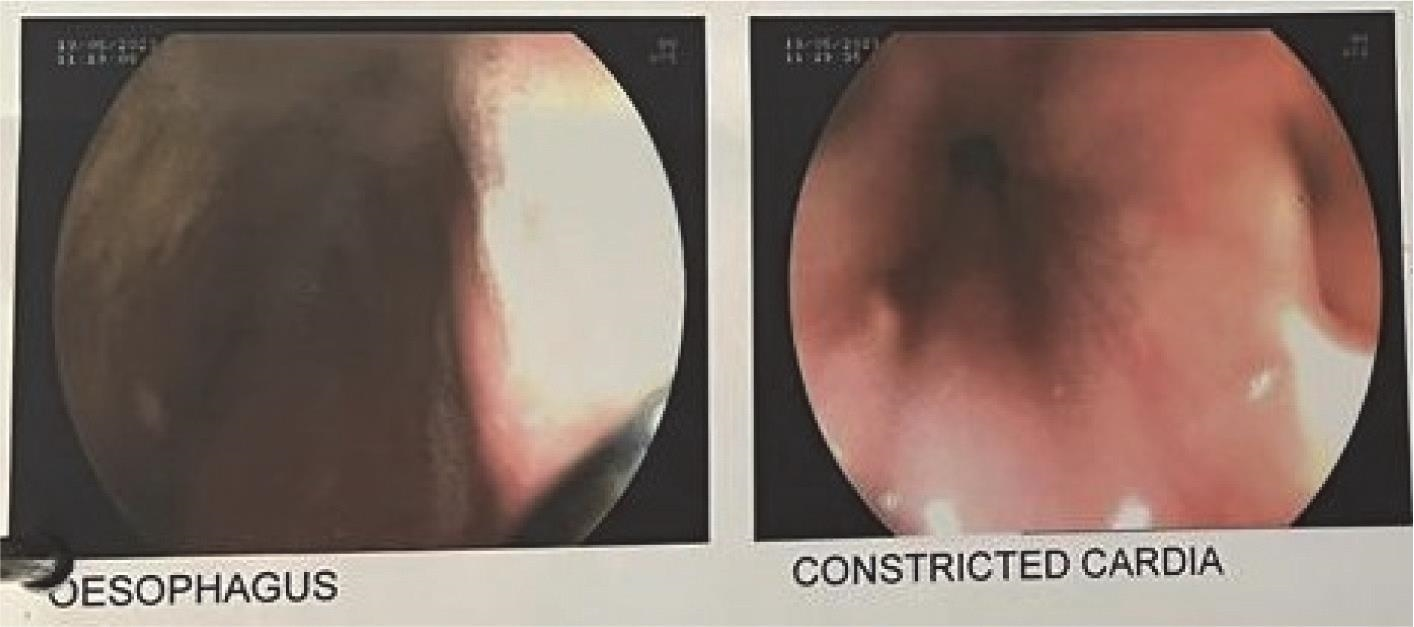
Upper GI endoscopy showing constricted cardia.

Initial upper gastrointestinal endoscopy showed a dilated esophagus filled with old food particles with constriction at the cardia (Figure 1).

**Figure 2:**
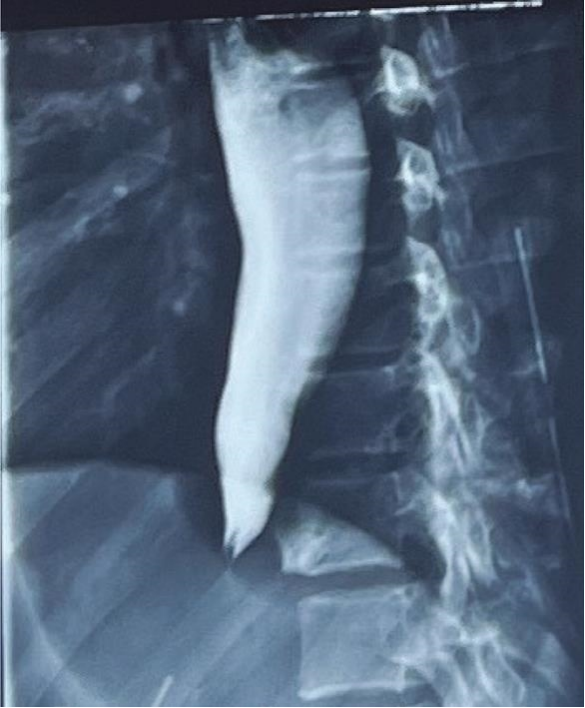
Initial barium swallow.

Barium swallow showed a classical bird's beak sign, that is, narrowing at the terminal esophagus, with dilation above the narrowing (Figure 2).

Manometry was not done due to test unavailability in Uganda.

### 2.4. Diagnosis

She was diagnosed with achalasia cardia based on the characteristic clinical history of progressive dysphagia, supported by the findings on endoscopy and barium shallow.

### 2.5. Treatment

On admission, our patient received initial emergency care which included fluid resuscitation and correction of electrolyte imbalances. After stabilization, we were able to perform diagnostic tests (upper gastrointestinal endoscopy and barium swallow) 2 days postadmission, which suggested achalasia cardia.

The patient was planned for Heller cardiomyotomy. A written informed consent was obtained from the patient and her mother in accordance with hospital policy; procedure risks, benefits, and alternative options were discussed in detail.

Heller cardiomyotomy via an open transthoracic approach was performed. The procedure involved a left anterolateral thoracotomy, with access obtained through the sixth intercostal space. A single cardiomyotomy incision was made ~4 cm distal and 6 cm proximal to the esophageal stenotic region. We then incised and separated longitudinal and circular muscles with resultant exposure of mucosa of the esophagus plus the proximal stomach. Belsey Mark IV fundoplication was then done as an antireflux closure technique.

### 2.6. Follow-Up and Outcome

She was admitted into the intensive care unit for 24 h after surgery for close monitoring, after which she was transferred to the main ward once stable. She was also able to start oral sips 24 h after surgery with great improvement in swallowing, and she was allowed home day 8 postsurgery.

**Figure 3:**
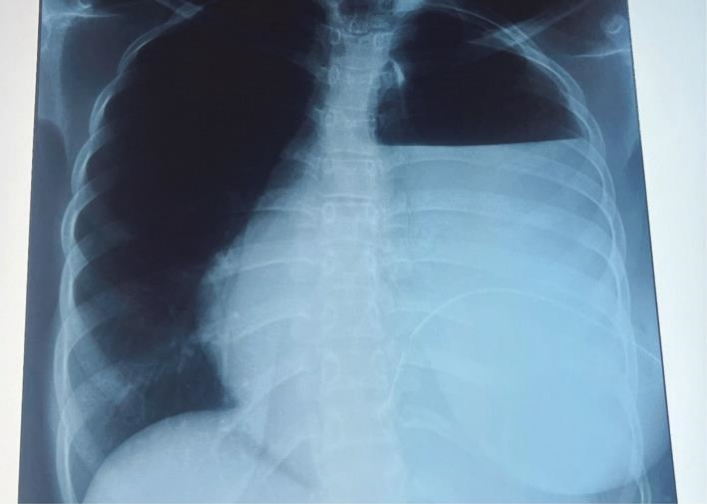
Chest X-ray showing left-sided pyothorax.

However, 22 days after surgery, she was readmitted with complaints of pus discharge from the thoracotomy incision site, fever, and difficulty in breathing for 3 days. Chest radiography revealed a homogenous opacity of the left hemithorax, involving the left lower and part of the upper lung zones (Figure 3). Left tube thoracostomy was performed with underwater seal drainage and draining pus.

Repeat barium swallow showed contrast medium spillage into the left chest cavity, suggesting esophageal perforation. A subsequent exploratory thoracotomy was performed, which confirmed an esophageal perforation at the cardiomyotomy site. During the procedure, the esophageal perforation was repaired, a jejunostomy feeding tube was placed, and the chest tube was removed followed by the creation of a pleurocutaneous window.

She was discharged from hospital 3 months after the second admission, with marked improvement in her nutritional status and a significant reduction in pus drainage from the pleurocutaneous window.

On subsequent follow-up, 1 month postdischarge (5 months post-Heller cardiomyotomy), the pleurocutaneous window had spontaneously closed, she reported normal swallowing, and her nutritional status had improved, evidenced by a weight of 46 kg and BMI of 18.7 kg/m^2^.

Our patient was last reviewed about 9 months post-Heller cardiomyotomy; she reported normal swallowing and complete resolution of all achalasia symptoms.

## 3. Discussion

### 3.1. Demographics and Etiology

Achalasia cardia is a relatively rare condition with a prevalence of ~10 cases per 100,000 people [2, 3, 7]. Although this disorder can affect all age groups, it is most commonly diagnosed in adults aged 25–60 years, with only about 5% cases noted among children and adolescents under 15 years of age [2, 3, 7, 8], and no significant sex predominance [2, 7].

### 3.2. Clinical Presentation

The most common presenting symptom is progressive dysphagia, initially with solids and later progressing to liquids. Additional symptoms may include regurgitation of feeds or postprandial vomiting, chest pain, weight loss, features of aspiration, nocturnal cough, hiccups, or difficulty belching [4, 9].

Our patient was an 18-year-old female who presented with classic features including dysphagia, postprandial vomiting, and significant weight loss.

### 3.3. Investigations and Diagnosis

During diagnostic workup for achalasia cardia, clinical history and examination findings are complemented by upper gastrointestinal endoscopy, barium swallow, and esophageal manometry [10, 11].

Upper gastrointestinal endoscopy is vital to exclude other benign causes of dysphagia and malignant causes of dysphagia and lower esophageal obstruction. Findings in achalasia may include a dilated or tortuous esophagus with saliva and/or food retention, as well as a tight esophageal gastric junction; no visible cause of esophageal obstruction is noted [10–12]. However, due to the low sensitivity of endoscopy, especially in early disease, it yields normal findings in nearly 40% of the patients [12].

Barium swallow is an invaluable primary diagnostic tool in low- and middle-income-income countries (LMICs) because it is more available and less expensive. The classic finding in esophageal achalasia is what is known as the bird's beak appearance, a dilated esophagus with progressive narrowing, as well as lack of peristalsis [10, 11]. Barium swallow may show normal findings in nearly 40% of patients with achalasia [13].

The gold standard for the diagnosis of achalasia remains high-resolution esophageal manometry [10, 11]; which reveals the three major criteria including aperistalsis of the esophageal body, absent or delayed relaxation of LES, and its elevated resting pressure [12]. However, manometry is often unavailable in LMICs, for example, Uganda, and the reliance on barium swallow for diagnosis persists, often resulting in delayed or missed diagnoses [10–13].

In our patient, the diagnosis of achalasia was based on clinical suspicion and complimented by typical findings on upper gastrointestinal endoscopy and barium swallow.

### 3.4. Treatment

The primary goal of management is to ease the symptoms of achalasia by reducing the outflow resistance caused by a nonrelaxing esophageal sphincter. Treatment modalities, including pharmacological, endoscopic, and surgical approaches, depend on local hospital capabilities, the patient's age, comorbidities, or preference [14].

The most effective therapies are currently considered to be pneumatic dilation and surgical myotomy [3, 5, 14]. Pneumatic dilation involves employing air pressures to disrupt the LES circular muscle fibers, while surgical myotomy involves the division of the circular muscle fibers of the LES, via open thoracotomy, laparotomy, or laparoscopy.

Our patient was treated with open Heller cardiomyotomy, a form of surgical myotomy. Although laparoscopic Heller cardiomyotomy is the recommended treatment modality in adolescents with achalasia cardia [5, 14], it is not available in our setting. An antireflux procedure, fundoplication is usually done to minimize the risk of gastroesophageal reflux after the procedure, which is one of the complications of surgical myotomy [14, 15]. In our patient, a Belsey Mark IV fundoplication as an antireflux closure technique was done.

For adolescents like our patient, pharmacological therapies, for example, calcium channel blockers or long-acting nitrates, are less effective as monotherapy [14, 15]. Peroral endoscopic myotomy, self-expanding metal stents, or endoscopic sclerotherapy procedures [14] are not yet available in the Ugandan setting.

### 3.5. Complications of Treatment

Esophageal perforation is a rare but possibly fatal complication of surgical treatment for achalasia cardia [6, 16]; it can occur intraoperatively, early or late in the postoperative period. To rule out this complication, it is recommended to perform a repeat barium swallow after the surgery [14, 15]. Our patient developed esophageal perforation after undergoing open Heller cardiomyotomy. However, this esophageal perforation was diagnosed 3 weeks after surgery when she presented with features of a left pyothorax.

Esophageal perforation, if recognized early, especially if small and asymptomatic may be managed conservatively with strict parenteral nutrition, gastric/esophageal aspiration, and broad-spectrum antibiotics [15]. In the case of large esophageal perforations or delayed identification of the perforation with subsequent mediastinal or pleural contamination, surgical procedures such as thoracostomy with esophageal repair, draining of the neighboring abscess, insertion of an esophageal stent, or insertion of a feeding jejunostomy may be required [14, 15, 17]. In our patient, the esophageal perforation resulted in a left pyothorax (pleural contamination) which was managed by chest tube insertion and open exploratory thoracotomy with subsequent esophageal repair and insertion of a feeding jejunostomy.

Other significant complications include achalasia recurrence or gastroesophageal reflux, among others [14–16]. Our patient reports normal swallowing and complete resolution of all achalasia symptoms 9 months post-Heller cardiomyotomy.

A preprint has previously been published in Authorea [18].

## 4. Conclusion

This case highlights the challenges in diagnosing and managing achalasia cardia, particularly in resource-limited settings like Uganda.

Given the rarity of achalasia, clinicians must maintain a heightened level of suspicion to detect potential cases, more so in adolescents and children who fall outside of the typical age range.

Based on our experience, patient education and obtaining formal consent are vital steps before initiating surgery or any treatment intervention. Even rare complications, such as the potential for esophageal perforation following surgical treatment of achalasia, should be thoroughly discussed with the patient.

In addition, patients can achieve favorable outcomes with the resolution of achalasia symptoms despite complications such as esophageal perforation following surgical myotomy, provided prompt management of these complications.

Furthermore, this case emphasizes the need for increased accessibility to advanced diagnostic tools like esophageal manometry in low-resource settings to enhance early detection and optimize patient care.

## Data Availability

Data are available on request from authors.
